# A new navigational apparatus for fixation of acetabular posterior column fractures with percutaneous retrograde lagscrew: Design and application

**DOI:** 10.1097/MD.0000000000012134

**Published:** 2018-09-07

**Authors:** Pijun Zhang, Jie Tang, Yonghui Dong, Lu Lu, Shengjie Wang, Shifeng Song, Gang Wang

**Affiliations:** a Department of Orthopedics, The Second Affiliated Hospital of Hainan Medical University, Hainan Medical University, Haikou, Hainan; b Department of Orthopedics, Henan Provincial People's Hospital, Zhengzhou, Henan; c Department of Orthopedics and Traumatology, Nanfang Hospital, Nnanfang Medical University, Guangzhou, Guangdong, People's Republic of China.

**Keywords:** 3D, acetabulum, bone screw, guide apparatu

## Abstract

The purpose of this study was to analyze the feasibility and accuracy of a newly developed guide apparatus for the percutaneous retrograde lag screw fixation of posterior column of acetabular fractures. 3D pelvic models were reconstructed from the helical computed tomographic data of 33 adult patients using the Mimics 10.01 software. The virtual cylindrical implants were placed along a line passing through the central point of the ischial tuberosity and the midpoint between the most prominent point of anterior superior iliac spine and that of posterior superior iliac spine. Some anatomical parameters were then measured, based on which a guide apparatus was developed, and its safety and accuracy were experimentally validated with pelvic and cadaveric specimens. The screws were successfully placed in all of the 66 hemipelves. There was a significant difference between the male and female groups in the AB distance (156.26 ± 7.28 mm and 151.38 ± 8.11 mm), OI distance (139.53 ± 7.56 mm and 125.15 ± 11.17 mm), and diameter (12.19 ± 1.97 mm and 10.19 ± 2.14 mm) of the virtual cylindrical implants. This guide apparatus was proved effective for percutaneous retrograde lag screw fixation of posterior column acetabular fractures by the experiments with the pelvic and cadaveric specimens. Screw fixation of posterior column fractures via OI is safe and feasible. We designed a new percutaneous retrograde screw fixation guide apparatus to assist internal fixation of posterior column acetabular fracture.

## 1 Introduction

Open reduction and internal fixation (ORIF) has proven to be effective in the treatment of posterior column acetabular fracture. However, because of the deep anatomical location of the acetabulum and its complex anatomical relationship with adjacent structures, the operation requires a long incision and wide exposure on the acetabulum, which is technically challenging. The intrinsic invasive nature of ORIF exposes patients to risks of hemorrhage and a number of postoperative complications, such as heterotopic ossification,^[1]^ iatrogenic sciatic nerve injury,^[2]^ and femoral head necrosis^[3]^ that are known to have an adverse effect on joint function.^[4,5]^ Reinert et al^[6]^ previously reported that lag screw fixation of a complex acetabular fracture allowed a small incision to be made, thus resulting in a good or excellent reduction of acetabulum in all patients. It was also reported to have a better biomechanical strength than internal fixation with a plate.

Recently percutaneous lag screw fixation has gained popularity as a minimally invasive treatment of acetabular fractures, such as the sacroiliac fracture or dislocation, anterior or posterior column fracture, and iliac crescent fracture.^[7–9]^ The advantage of this minimally invasive technique is to reduce bleeding and treatment costs. However, there is substantial evidence to suggest that percutaneous lag screw fixation increases the death caused by screw induced injury of large pelvic vessels, nerve or pelvic organ injuries, poor location or fixation, and screw fracture.^[10]^ The wider application of this approach is limited to a certain extent by the technical difficulties in determining the optimal entry point and direction, and the diameter of the screw, among others. The image-guided orthopedic surgery has the potential to be a widely used, minimally invasive, and intelligent option,^[11,12]^ which in fact has been increasingly practiced by orthopedic surgeons.^[13,14]^ Despite its therapeutic benefits, the application of this technology in most Chinese hospitals is limited, mainly because of the high cost of equipment and materials. ^[10]^

In this study, the virtual 3D pelvic models were reconstructed using the Mimics 10.01 software and lag screw placement was simulated, based on which a guide apparatus was developed for percutaneous retrograde lag screw fixation of posterior column acetabular fracture, and its safety and accuracy were assessed with pelvic specimens.

## 2 Materials and methods

### 2.1 Data collection and reconstruction of 3D pelvic models

The complete helical CT data were collected from 33 adult patients (15 males and 18 females aged from 15 to 77 years, 45.6 ± 18.5) without any bone lesions or anatomic abnormalities from November 2014 to November 2016. The study was approved by our hospital's institutional review board, and all the patients provided informed consent before participation. The CT scanning was performed using a GE Medical systems/Light speed 16 scanner under the conditions of 120 kV, slice thickness = 1.25 mm, and 512 × 512 matrix. The CT data were then imported in DICOM format into the Mimics 10.01 software (Materialise, Leuven, Belgium). Firstly, the pelvic region was extracted from the CT images by the gray-level thresholding, then the bilateral pelvic boundaries were precisely extracted by the region segmentation, and finally the 3D pelvic models were reconstructed.

### 2.2 Determination of the screw entry point and anatomic parameters in Mimics

**Figure 1:**
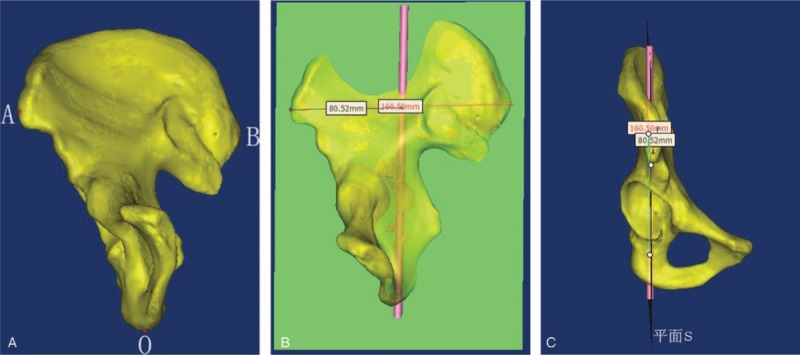
Placement of a virtual cylindrical implant. (A, B) The most prominent point of anterior superior iliac spine and that of posterior superior iliac spine. (C) The central point of the ischial tuberosity.

The 3D bilateral pelvic models were reconstructed using the Mimics 10.01 software (Fig. 1). A virtual cylindrical implant of about 6.5 mm in diameter was placed along the medial axis (OI) passing through the central point of the ischial tuberosity (O) and the midpoint (I) between the most prominent point of anterior superior iliac spine (A, ASIS) and that of posterior superior iliac spine (B, PSIS), where O was the screw entry point and I was the end point of the guide apparatus. A plane (S) was formed by the three points of A, B, and O. The distance between A and B was measured, and the length of the lag screw planted was determined by the distance between the entry (O) and exit point (i) at the lower part of the iliac fossa, or at the outer table of the ilium and above the greater sciatic notch, from which the virtual cylindrical implant came out. However, the cases in which i was located below the greater sciatic notch were considered as a failure. The results would be used to determine the feasibility of the retrograde lag screw fixation of posterior column acetabular fracture. The diameter of the virtual cylindrical implant was increased by 0.1 mm at each increment, and the maximum diameter (d) was the diameter of the implant that could not penetrate into the joint (Fig. 1).

### 2.3 Measurements of the anatomic parameters of acetabulum and design of the 3D guide apparatus

**Figure 2:**
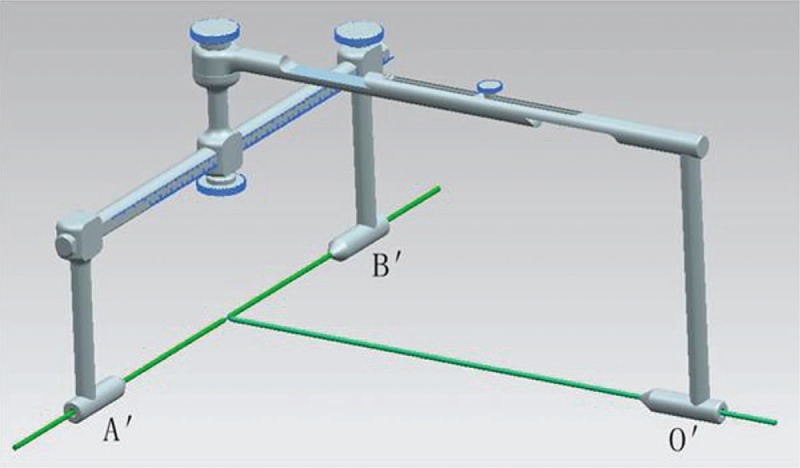
A design model of the guide apparatus (A′, B′ and O′ are corresponding to A, B, and O, the slender bars are the kirschner pins).

There was a significant difference in the AB distance (156.26 ± 7.28 mm and 151.38 ± 8.11 mm, *t* = 2.574, *P* < .05), Oi distance (139.53 ± 7.56 mm and 125.15 ± 11.17 mm, *t* = 5.992, *P* < .05), and diameter (12.19 ± 1.97 mm and 10.19 ± 2.14 mm, *t* = 3.923, *P* < .05) of the virtual cylindrical implants between the male and female groups (Table 1). The screws were successfully placed in all of the 66 hemipelves without penetration into the joint. All these results indicated that it was feasible for screw fixation of posterior column acetabular fracture through a line OI that passing through the central point of the ischial tuberosity and the midpoint between ASIS and PSIS. A guide apparatus was then developed (Fig. 2).

**Table 1:**
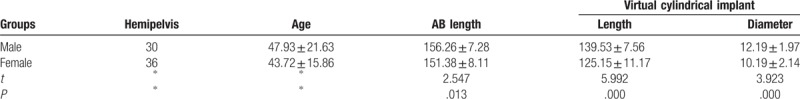
Anatomical parameters of the percutaneous retrograde lag screw fixation of posterior column acetabular fracture (mm, X ± s).

### 2.4 Verification with the iliac specimens

**Figure 3:**
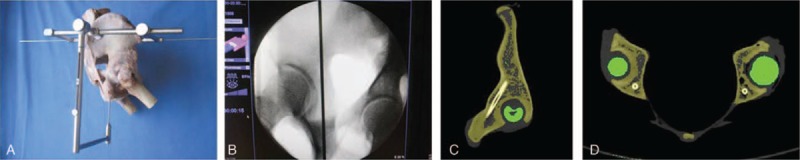
(A) An effect drawing of screw fixation in the ilium. (B) Oblique film. (C) Sagittal computed tomography (CT). (D) Axial CT (lag screws of 10 cm in length and 7.3 mm in diameter in the posterior column, as indicated by the red arrow).

**Figure 4:**
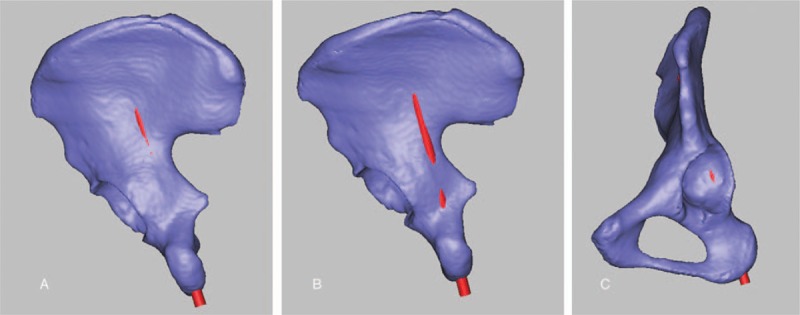
(A) Excellent placement of the virtual cylindrical implant. (B, C) Failed modes (B. Running out of the posterior column, C. Penetrating into the joint).

It was evaluated with 18 adult unilateral iliac specimens (9 males and 9 females). The A′-end of the guide apparatus was fixed to the most prominent point of ASIS with kirschner pins, and the B′-end to that of PSIS, respectively. A sliding bar between the A′- and B′-end was used to measure the distance between ASIS and PSIS. The O′ bar was adjusted to the midpoint of AB′ and then fixed with a bolt. The guide apparatus was maintained stable throughout the surgical procedure with the help of an assistant. A kirschner pin was drilled into the ilium via the central point of the ischial tuberosity guided by the sleeve of the guide apparatus (Fig. 3), and the exit point of the kirschner pin was observed. Clearly, there were 3 possibilities for the exit points: at the iliac fossa; at the outer table of the ilium and above the greater sciatic notch; or (3) below the greater sciatic notch, such as penetration into the acetabulum or the tetragonal areas, which were rated as excellent, good, or failed in this study, respectively (Fig. 4). The diameter of the kirschner wire used in this experiment is 2.5 mm, and the diameter of the screw used is 6.5 mm. The success rate and screw length were recorded, and the difference between the male and female groups was compared.

### 2.5 Mimics simulation of the screw fixation in the ilium

**Figure 5:**
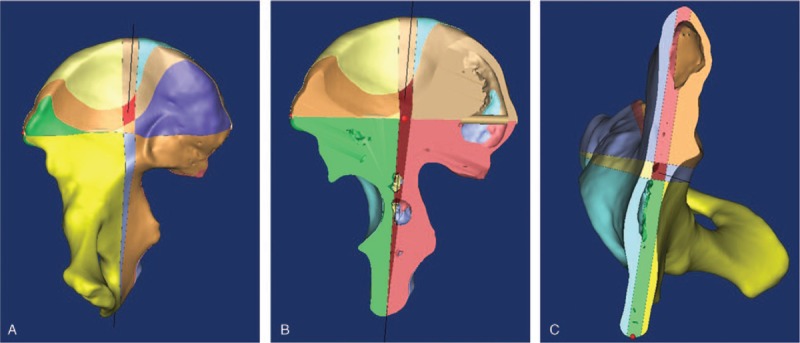
Safety verification of the screw fixation in the ilium. (A) Medial view. (B) Longitudinal profile. (C) Horizontal profile (the dark red area is the testing area).

ASIS and PSIS are the bony projection of the pelvis that protrude at the front and back, which can be difficult to be precisely fixed with kirschner pins in real surgical practice. The location errors were evaluated with the 33 reconstructed models of the right hemipelves in the Mimics software. A plane of 250 × 150 mm was created by the most prominent points of ASIS and PSIS and the central point of the ischial tuberosity. A virtual cylinder of 6.5 mm in diameter was placed along the line connecting ASIS and PSIS as the lateral axis, and the line passing through the central point of the ischial tuberosity and the midpoint of the lateral axis was created as the medial axis. A sphere of 8 mm in diameter was placed at the cross point of the 2 axes. The medial axis of the implant was set close to the medial edge of the lateral axis, and moved from left-to-right or vice versa within the range of the sphere diameter (±4 mm), and the lateral edge was examined in a similar manner (Fig. 5).

### 2.6 Safety of the screw entry point at the ischial tuberosity

**Figure 6:**
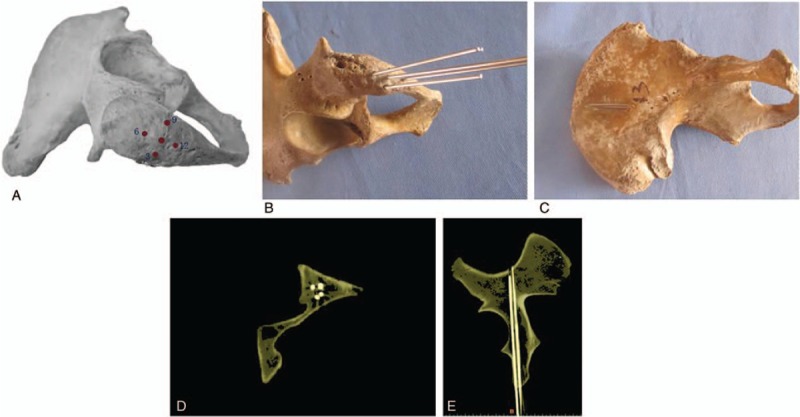
(A) Five screw entry points at the ischial tuberosity. (B, C) An effect drawing of screws placed. (D, E) Computed tomography revealed excellent screw position.

The ischial tuberosity has a wider range in comparison with the ASIS or PSIS, thus making it more difficult to determine the entry points in percutaneous screw fixation. In this study, we examined 4 screw entry points in the 18 hemipelves: at the 12 O’clock position (5 mm from the central point of the ischial tuberosity), 3 O’clock position (5 mm from the ischial edge), 6 O’clock position (5 mm from the central point of the ischial tuberosity), and 9 O’clock position (5 mm from the ischial edge) of the central point of the ischial tuberosity (Fig. 6).

### 2.7 Feasibility of the screw fixation guided by the guide apparatus in the cadaveric specimens

**Figure 7:**
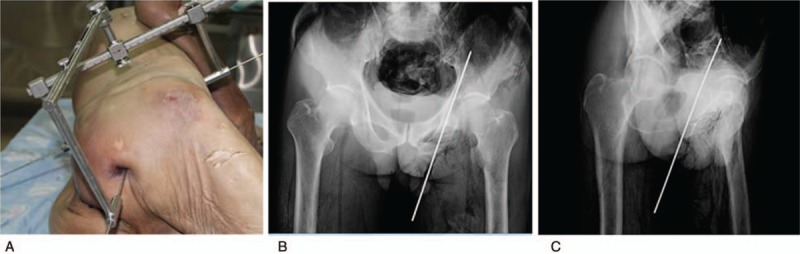
(A) Fixation of the guide apparatus to the cadavers in a lateral position. (B, C) Pelvic anteroposterior and iliac oblique x-ray revealed excellent screw position.

The surgical site is to a certain extent obscured by the soft tissues in a real surgical setting, which would introduce additional difficulties to the use of the guide apparatus. Then the kirschner pins were placed in 2 adult (1 male and 1 female) fresh frozen cadavers at the anatomy laboratory of the Southern Medical University. The soft tissues were completely thawed before the experiments. The cadavers were placed in a lateral position, and the surface locations of ASIS, PSIS, and ischial tuberosity were marked. A longitudinal incision of about 1.0 cm and 1.5 cm was made at the marked site of ASIS and PSIS, respectively, and then the subcutaneous soft tissues were stripped away to expose the most prominent point of ASIS and PSIS, to which the guide apparatus was fixed with a 2.0-mm kirschner pin. The AB length between ASIS and PSIS was determined by the transverse bar, and then the longitudinal bar was adjusted to the midpoint of AB′ and then fixed with a bolt. The guide apparatus was maintained stable throughout the surgical procedure with the help of an assistant. The lower limb on the operation side was maintained a position of hip extension and knee flexion. A incision of about 5.0 cm was made at the marked site of the ischial tuberosity, through which the sleeve of the longitudinal bar was inserted by about 2.5 mm to the central point of ischial tuberosity, then the longitudinal bar was locked by a bolt at the end of the bar. As the C-shaped arm showed that the ischial tuberosity and the oblique-iliac crest were accurately located, the kirschner pins were drilled by the electric drills. The accuracy of screw position was determined by postoperative x-ray images (Fig. 7).

### 2.8 Statistical analysis

Data are presented as means standard ± deviation. Statistical analyses included a 2-tailed paired *t* test to assess differences between 2 groups. Between-group comparisons for all analyses were performed with analysis of variance. Significance was set at *P* < .05 for all tests.

## 3 Results

This experiment was carried out with 18 adult iliac specimens (9 males and 9 females). The results were rated as excellent in 15 cases (83%) and good in the rest 3 cases (17%), and the male specimens could accommodate a longer screw than the female specimens (131.53 ± 5.36 mm and 118.15 ± 9.03 mm, respectively, *P* < .05) (Table 2).

**Table 2:**
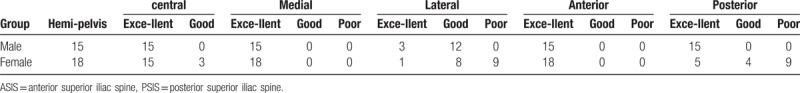
The safety of the screw fixation at the ASIS and PSIS.

Among the 15 male hemipelves underwent screw fixation, the results were rated as excellent in all cases for screw fixation at the medial, anterior, and posterior side, whereas excellent in 3 (20%) and good in 12 cases (80%) for that at the lateral side, respectively. Of the 18 female hemipelves underwent screw fixation, the results were rated as excellent in all cases for screw fixation at either medial or lateral side; excellent in 1 (6%), good in 8 (44%), and poor in 9 (50%) cases for that at the lateral side; and excellent in 5 (28%), good in 4 (22%), and poor in 9 (50%) cases for that at the posterior side, respectively. It showed that the entry point was preferably set at the medial side of ASIS and PSIS, or at a point deflected slightly to ASIS (Table 2).

The results were rated as excellent in 15 and good in 3 cases for the screw fixation at the medial, lateral, and anterior side, which was consistent with that at the central point. However, for screw fixation at the posterior side, the results were rated as excellent in 11, good in 3, and failed in 4 cases, respectively. The typical failure mode was the penetration at the lower acetabulum and above the ischial tuberosity. It can be concluded that the ischial tuberosity has a large area suitable for screw entry point. In general, a satisfactory result would be obtained except a too posterior position was selected. In addition, the result would be better for those with a large pelvis.

Only 2 intraoperative x-ray views were required, which revealed excellent screw position in the pelvis, indicating that this guide apparatus could be a valuable clinical tool.

## 4 Discussion

There are a variety of internal fixation techniques for acetabular fractures. The most common method is to use 6 or 8 hole plates on the anterior column or post column. ORIF can provide effective reduction and internal fixation, but it may increase the risk of intraoperative and postoperative haemorrhage. The increased risk is because of the fact that it often requires long incisions and sufficient exposure of the acetabulum to fix the plate. Although the patient has taken a position of hip extension and knee flexion, and great care was taken to avoid sciatic nerve injury, the incidence of sciatic nerve injury was reported to be between 2% and 6%.^[2]^ Intraoperative real-time EMG monitoring has been advocated during acetabular fracture repair and complex total hip arthroplasties to prevent iatrogenic sciatic nerve injury.^[15]^ However, it seems not to be reliable enough, as Haidukewych reported that the using of intraoperative real-time EMG monitoring did not decrease the rate of iatrogenic sciatic palsies.^[16]^

Possible injuries pertinent to the anterior approach include including femoral nerve injury and lateral femoral cutaneous nerve injury, whereas that pertinent to the posterior (K-L) approach include superior or inferior gluteal nerve injury, which would result in postoperative gait dysfunction. Neal et al^[17]^ reported that the incidence of any heterotopic bone formation was 51% after acetabular fracture repair and the incidence of severe heterotopic bone formation was 9% to 19%. It was found that the postoperative heterotopic ossification occurred in about 85% unmonitored patients by the extended iliofemoral approach,^[16]^ and in about 40% to 55% unmonitored patients by the K-L approach.^[18]^ At least 20% patients with heterotopic ossification would have hip joint dysfunction.^[19]^ However, either approach to treat acetabular fractures can affect the blood supply of the femoral head and increase the risk of femoral head necrosis.^[20]^ In contrast, the minimally invasive treatment seems to be more effective in decreasing the rate of iatrogenic sciatic nerve injury and postoperative heterotopic ossification, minimizing the stripping range, protecting the blood supply of femoral head, and ultimately decreasing the rate of femoral head necrosis. Charles et al suggested that a transverse acetabular fracture could be fixed by 2 screws, one to the posterior column and the other one to the anterior column.^[6]^ It was found that there was no significant difference between lag screw fixation and plate fixation in terms of biomechanical stability.^[21]^ Gaye first reported that CT guided percutaneous screw fixation for acetabular fractures is suitable for small fractures of the dome or posterior column.^[22]^ Thus, percutaneous retrograde lag screw fixation is considered as an optimal treatment option for acetabular fracture, with the advantages of minimal surgical trauma, short operation time, little bleeding, and percutaneous treatment.

A previous study indicated that the maximum diameter of the virtual implant accommodated by the anterior or posterior column was much smaller than the smallest diameter of the columns; thus, the size of the screws used for percutaneous fixation of acetabular fractures should not be based solely on the measurement of cross-sectional diameter of the columns.^[23]^ Zhang et al previously proposed that the most prominent points of ASIS and PSIS were equidistant from a point at the ischial tuberosity, based on which they further proposed that the lag screws might be placed percutaneously through the perpendicular bisector of a line passing through the most prominent points of ASIS and PSIS.^[20]^ Based on Zhang's study, the 3D pelvic models were reconstructed using the Mimics 10.01 software and percutaneous retrograde screw fixation of posterior column acetabular fracture was simulated in this study. The results showed that a virtual cylindrical implant could be placed along the line passing through the central point of the ischial tuberosity and the midpoint between ASIS and PSIS. Adding a small incision did not expose the patient to a higher risk of complications.

The guide apparatus described in this study has several advantages. First, it consists of 2 halves of C-shaped frame joined together by a screw, as shown in Figure 2. The screw position is defined by the intersection line of the 2 planes. Second, the frame-style apparatus is rigidly fixed to the pelvis, which provides a stable reference that will not change with the body position of the patient. Third, there are 2 sets of scales at the sliding bar for the measurement of the distance between A′ and B′, it is easy for the O′ bar to be precisely located at the midpoint of A′B′. Fourth, it can be elongated as desired when applied to patients with different somatotype. Fifth, the midpoint between ASIS and PSIS can be determined extracorporeally, through which the screw guided by this apparatus is bound to pass.

Traditional percutaneous retrograde screw fixation of posterior column fractures often requires repeated attempts to achieve normal reduction and fixation. It is difficult to put screws in precise positions, which will significantly increase the operation time. A major drawback associated with repeated operation is the bone injuries at a fixed position, which will have a detrimental effect on the adjustment of screw direction and the final fixation strength. CT-guided minimally invasive surgery, although accurate, has often been frustrated in many hospitals primarily because of the high cost. This new guide apparatus proposed in this study has the potential to become an alternative option, and proved to be effective by the Mimics simulation and experiments with pelvic and cadaveric specimens. It is safe and applicable to both male and female patients. It also has the advantages of high one-time success rate, long screw, simple structure, easy manipulation, small size, easy assembling, transportation, and sterilization. It allows for a minimal incision just large enough for the guide apparatus to be inserted, which as a result can reduce the pain of patient and reduce the hospitalization cost. The new guide apparatus is suitable for patients with acetabular posterior column fracture without displacement, or displacement ≤1 mm. Because it is difficult to palpate the ASIS, PSIS, and ischial tuberosities, the navigational apparatus is not recommended for use in obese patients.

## 5 Conclusion

A new guide apparatus was designed for percutaneous retrograde screw fixation of posterior column acetabular fracture using the Mimics software, which proved to be effective by the Mimics simulation and experiments with the pelvic and cadaveric specimens. In the next step, we will subject this guide apparatus to the clinical practice and assessment.

## Author contributions

**Conceptualization:** Shengjie Wang.

**Data curation:** Jie Tang.

**Formal analysis:** Yonghui Dong.

**Funding acquisition:** Bing Wang.

**Investigation:** Lu Lu.

**Methodology:** Shifeng Song.

**Project administration:** Pijun Zhang.

**Writing – original draft:** Pijun Zhang.

**Writing – review & editing:** Bing Wang.
